# Giant cavernous hepatic hemangioma shrunk by use of sorafenib

**DOI:** 10.1007/s12328-012-0343-0

**Published:** 2012-11-29

**Authors:** Satoyoshi Yamashita, Kohsuke Okita, Katsunori Harada, Atsuyoshi Hirano, Teruaki Kimura, Akira Kato, Kiwamu Okita

**Affiliations:** Department of Gastroenterology, Social Insurance Shimonoseki Welfare Hospital, 3-3-8 Kami-Shinchi, Shimonoseki, Yamaguchi 750-0061 Japan

**Keywords:** Hepatic hemangioma, Sorafenib, Transcatheter arterial embolization

## Abstract

Here we report a case of a 76-year-old man with a giant cavernous hepatic hemangioma of more than 20 cm in diameter. Since the hepatic hemangioma was actually growing and might possibly rupture and he complained of abdominal symptoms, we decided to perform interventional therapy. First we performed transcatheter arterial embolization (TAE) of the hepatic arteries. However, since this was not sufficiently effective, we added sorafenib (600 mg/day). As a result, the tumor shrank with symptomatic improvement. Subsequently, an adverse event occurred, and we suspended the sorafenib therapy. Then, the tumor began to grow, and we resumed administering sorafenib at 400 mg/day. The tumor shrank again, and we continued the sorafenib therapy thereafter. The tumor shrinkage, although possibly induced by the effect of TAE, is considered primarily due to the effect of treatment with sorafenib, because (1) TAE did not sufficiently reduce the blood supply to the inside of the tumor; (2) other tumors shrank in the area not targeted by TAE; and (3) the tumor grew during suspension of sorafenib therapy and shrank again after resuming the treatment.

## Introduction

Hepatic hemangioma is a benign vascular tumor often encountered in daily practice. Its incidence rate is estimated to be 1–2 %, and it is incidentally discovered in most cases on abdominal imaging studies [1, 2]. Most hemangiomas are less than 4 cm in diameter and remain asymptomatic, and very few cases require treatment. However, treatment is required if the tumor is growing with manifestation of abdominal symptoms, including abdominal pain, nausea, a feeling of abdominal distention, and anorexia, or if tumor hemorrhage or rupture occurs or may occur [3–6]. Surgical resection is reported to be effective for the treatment of hepatic hemangiomas [7–11]. In the cases of unresectable hemangiomas, however, radiation, ligation of hepatic artery, hepatic arterial embolization, or liver transplantation is selected.

Although the pathogenesis of hemangiomas is not clear, hemangiomas were previously thought to be congenital vascular malformation. In recent years, however, studies have suggested the possibility that abnormal angiogenesis induced by increases in vascular growth factors such as vascular endothelial growth factor (VEGF) is implicated in the pathogenesis [12, 13]. In addition, it has been reported that hemangiomas were shrunk by multikinase inhibitor which containing VEGF inhibitory activity [14]. Based on these findings, application of angiogenic inhibitors to the treatment of hemangiomas can be expected.

Here we report a case of a patient with a giant hepatic hemangioma in which sorafenib, a VEGF inhibitor, shrank the tumor. To our knowledge, this is the first report of a case in which sorafenib was administered to treat a hepatic hemangioma.

## Case report

A 76-year-old man visited our department as an outpatient for a detailed examination of an abdominal mass. At the age of 55, he was found, on a health check-up for employees, to have abnormal liver function tests. At his visit to a referral hospital, he was diagnosed as having a hepatic hemangioma. However, he ceased to visited the hospital and left the disease untreated. The tumor size at the time of the first diagnosis is unknown. Around the age of 75, he noticed an increase in waist circumference. Since abdominal distention progressed, he visited a practitioner in the neighborhood at the age of 76. Abdominal computed tomography (CT) revealed a large tumor involving the entire left lobe of the liver, and he was referred to our department.

At his first visit, he had a swollen upper abdomen due to the enlarged liver, and the lower edge of the liver reached 7–8 finger breadths below the xiphoid process. Blood test results (normal ranges) were as follows: peripheral white blood cell count, 4200/μL (3800–9800); peripheral red blood cell count, 434 × 10^4^/μL (430–550 × 10^4^); peripheral platelet count, 12.8 × 10^4^/μL (13.0–35.0 × 10^4^); aspartate aminotransferase, 29 U/L (10–38); alanine aminotransferase, 31 U/L (4–35); alkaline phosphatase, 231 U/L (100–340); γ-glutamyl transpeptidase, 128 U/L (11–64); total bilirubin, 1.49 mg/dL (0.2–1.0); C-reactive protein, 0.06 mg/dL (0–0.2); hepatitis B surface antigen, negative; hepatitis C antibody, negative; α-fetoprotein, 6.8 ng/mL (0–10); CA19-9, 9.1 U/mL (0–37); CEA, 2.0 ng/mL (0–5); PIVKA-II, 11 mAU/mL (0–40); VEGF, 23 pg/mL(0–115).

**Fig. 1:**
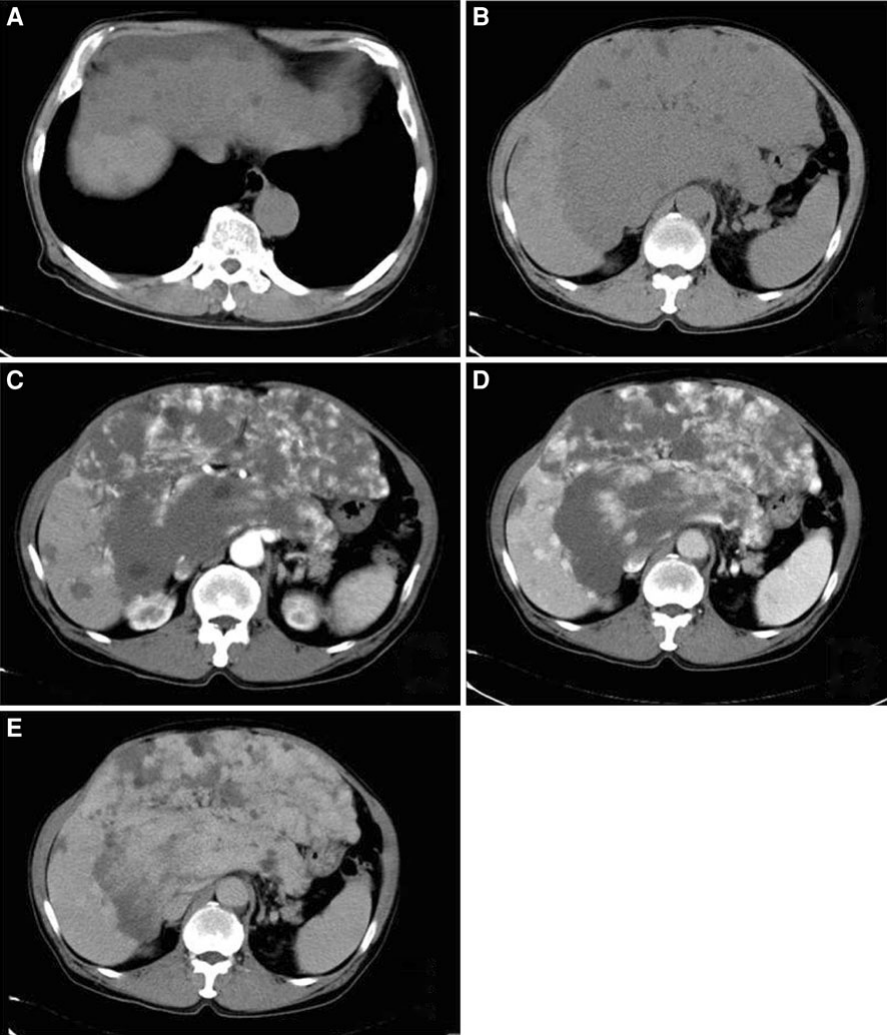
Dynamic CT of the abdomen with intravenous administration of contrast medium at the first visit. **a**, **b** Non-contrast CT; a large low-density area of more than 20 cm in the greatest diameter is observed in the entire left lobe and a large part of the right lobe, which pushed up the abdominal wall. It demonstrated subcapsule hematoma in some parts of tumor. Other than the primary tumor, multiple low-density areas of less than 2 cm in diameter are observed in the right lobe. The remaining tumor-free area of the liver is very small. **c** Contrast-enhanced arterial phase; the tumor is heterogeneously enhanced, with its margin enhanced first. **d** Contrast-enhanced portal venous phase; the contrast enhancement gradually spreads heterogeneously to the insider of the tumor. **e** Equilibrium phase; the entire tumor shows delayed staining without washout

Dynamic CT of the abdomen at his first visit (Fig. 1) revealed a large mass of more than 20 cm in the greatest diameter over the entire left lobe and a part of the right lobe of the liver. The image was consistent with hepatic hemangioma because the tumor was unequally stained in arterial phase, and the inferior portion of tumor was deeply delayed stained in late phase. It demonstrated multiple small nodules in right hepatic lobe with subcapsule hematoma in some parts of the tumor.

Since the tumor was growing and might be malignant in nature (e.g., angiosarcoma), percutaneous tumor biopsies were performed twice at an interval; however, an adequate amount of tissue could not be obtained and no definitive diagnosis was made. Since it was difficult to make a diagnosis based on liver biopsy, we performed dynamic CT during arteriography (CTA) instead.

**Fig. 2:**
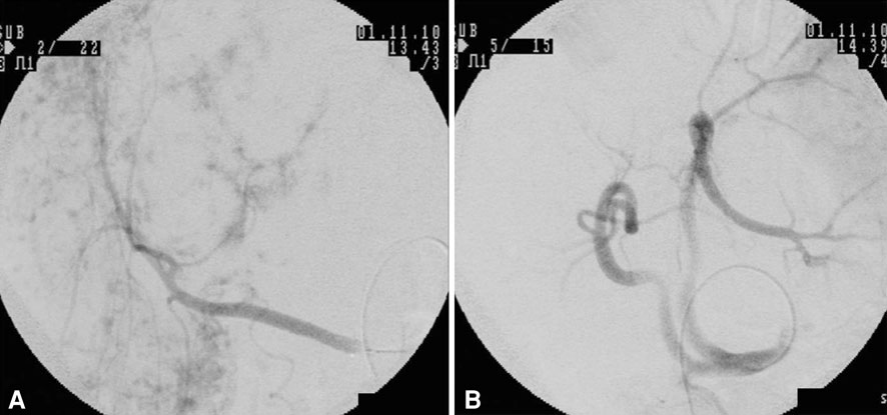
Hepatic arteriography. **a** Right hepatic arteriography; it was stained faintly and patchily, but not observed abnormal angiogenesis or irregularity. **b** Left and middle hepatic arteriography; abnormal angiogenesis or irregularity in the hepatic artery was not observed

**Fig. 3:**
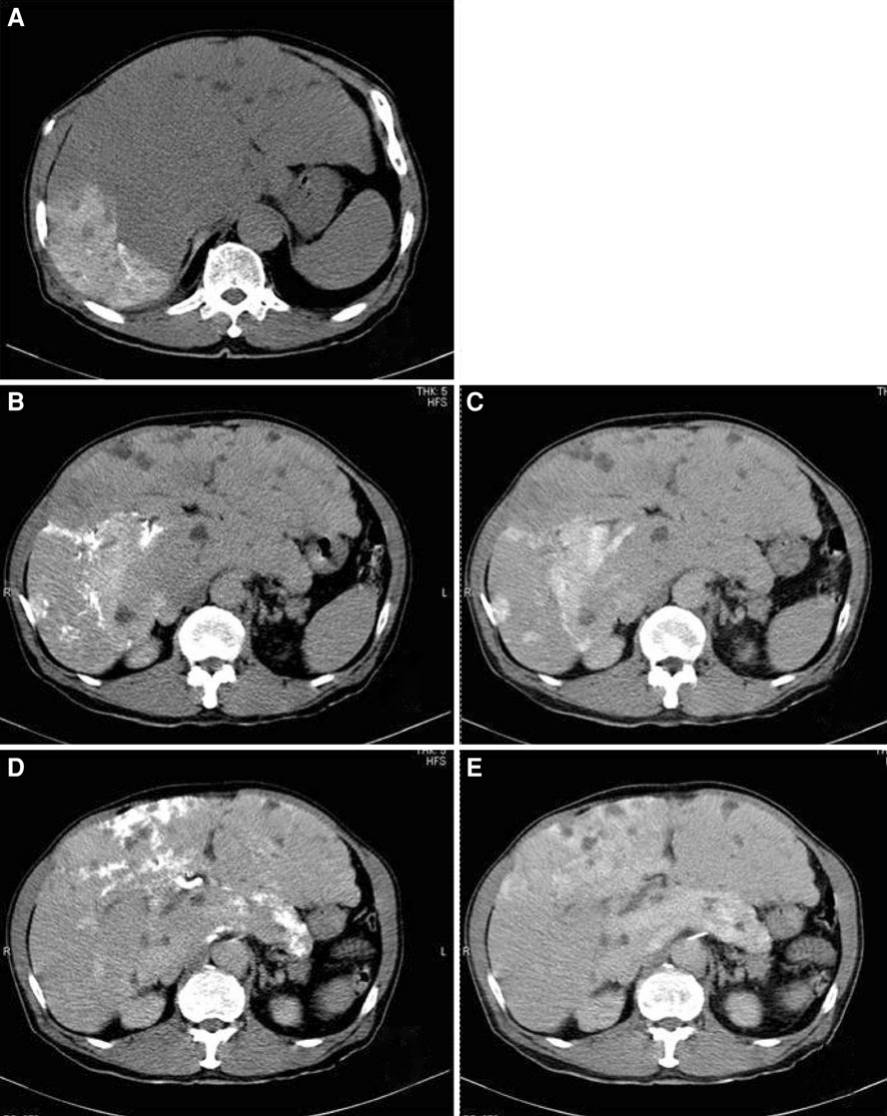
CTAP and CTA before the TAE. **a** CTAP; the site of the tumor exhibited a filling defect. **b** Early-phase CTA via the right hepatic artery; the tumors in the right hepatic lobe are heterogeneously enhanced. **c** Late-phase CTA via the right hepatic artery; the homogeneous enhancement remained in the tumors in the right hepatic lobe. **d** Early-phase CTA via the left and middle hepatic arteries; a part of the large tumor in the left hepatic lobe is heterogeneously enhanced. **e** Late-phase CTA via the left and middle hepatic arteries; the homogeneous enhancement remained in the entire tumor in the left hepatic lobe. Some parts of the tumor are not stained

The right hepatic artery stemmed from the superior mesenteric artery, and the middle and left hepatic arteries stemmed from the celiac artery. The hepatic arteriography showed no abnormal angiogenesis in all of right, middle and left hepatic artery and it showed light and finely-distributed with widespread and multiple image (Fig. 2). CT during arterial portography (CTAP) revealed a filling defect at the site of the tumor. On CTA, the mass showed early heterogeneous enhancement and residual enhancement in the late phase (Fig. 3).

On the basis of those CT images, cavernous hemangioma was diagnosed. We decided to treat this giant hepatic hemangioma based on increasing tumor size, abdomen enlarged feeling and risk of rupture because tumor existed on surface of liver with subcapsular ecchymoma.

**Fig. 4:**
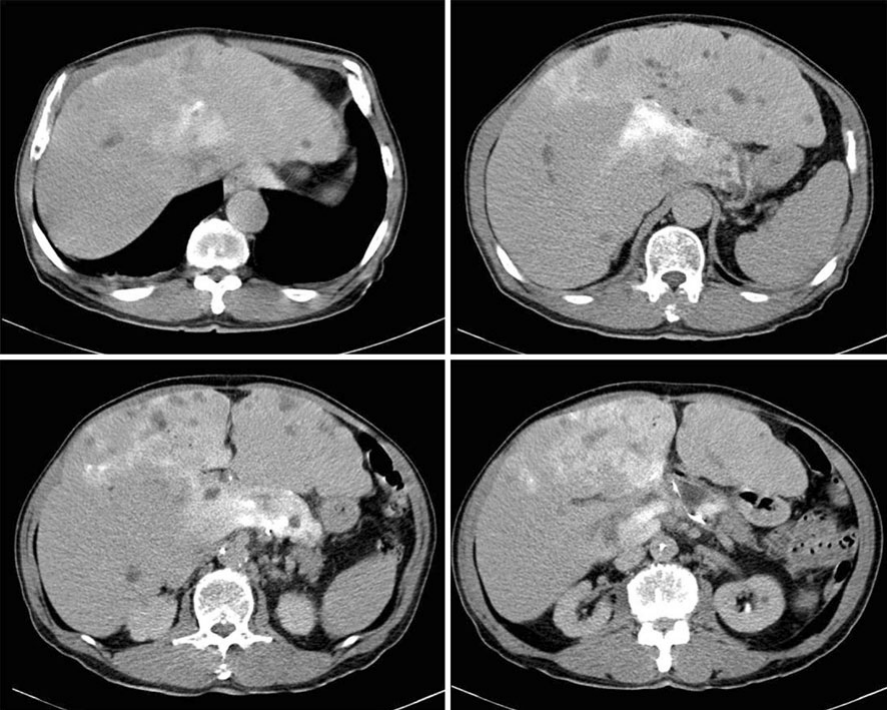
Non-contrast CT immediately after TAE Bright area showed pooled gelatin particles. The area of pooled gelatin particles was very limited compared to the total volume of primary tumor in left and middle hepatic lobe

We performed abdominal angiography followed by transcatheter arterial embolization (TAE). That is, 2 mg of contrast agent mixed suspension gelatin particles (Gelpart^®^; Nippon Kayaku Co. Ltd., Tokyo, Japan) [15] was injected from left hepatic artery and 2 mg from middle hepatic artery. We did not perform TAE from the right hepatic artery because the primary tumor was mainly fed by the left and middle hepatic arteies, and the tumor in right hepatic lobe was observed only as small nodules fed by right hepatic artery. Gelatin particles remained in just a part of tumor in non-contrast CT after performing TAE, and it validated as not effective (Fig. 4). Consequently, we considered adding another treatment.

In performing an additional TAE, we determined that repeated embolization was required in this case. However, Miyayama et al. [16] reported that four courses of chemoembolization with gelatin particles were not get enough efficacies for hepatocellular carcinoma (HCC) with 14 cm diameter. We were not sure to get enough efficacies by repeated TAE. Therefore we decided not to perform repeated TAE in this case. We did not make a choice of resection since the giant tumor size of hepatic hemangioma may increase a risk of hepatic failure after resection.

Therefore, we decided to initiate sorafenib used as molecular target agent for HCC. The dosing level of sorafenib was determined based on its standard dose in the treatment of HCC. In other words, while the standard dose is 800 mg/day, we chose a lower loading dose of 600 mg/day, allowing for the risk of adverse events in this elderly patient. We administrated sorafenib 600 mg/day orally 3 days after TAE (day 38) with appropriate informed consent and the approval of the internal institutional review board committee.

**Fig. 5:**
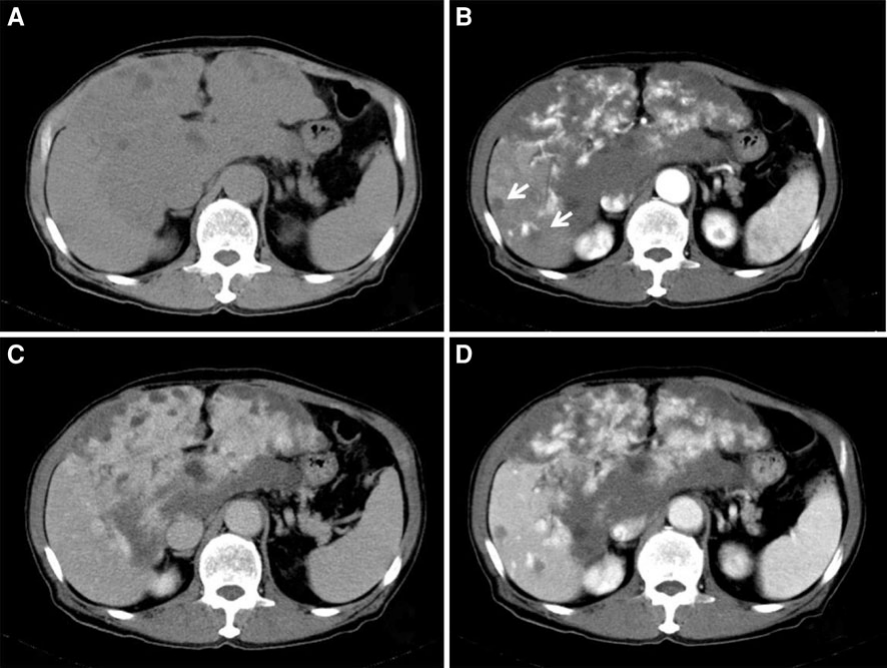
Dynamic CT of the abdomen with intravenous administration of contrast medium on day 31 of sorafenib therapy. **a** Non-contrast CT. **b** Contrast-enhanced arterial phase. **c** Contrast-enhanced portal venous phase. **d** Equilibrium phase. As compared with the baseline images; the size of the tumor in the left hepatic lobe is reduced, but the intensity of staining of the inside of the tumor is almost unchanged. In the right hepatic lobe also, on which TAE had not been performed, reduction in the tumor size was observed after treatment (11 mm in diameter, 13 mm; **b** indicated by *arrows*) as compared with the size before treatment (22 mm in diameter, 16 mm; Fig. 1c). Please note that the swollen abdominal wall observed in Fig. 1 has flattened

Tumor shrinkage was confirmed by abdominal CT on 31 days after sorafenib administration (day 68). We also confirmed tumor shrinkage in the tumor of the right hepatic lobe, on which TAE was not performed (Fig. 5). At that time, the lower edge of the liver was 5–6 finger breadths below the xiphoid process, and the tumor shrinkage was also confirmed by palpation. The patient was also aware of reduction of abdominal distention.

Diarrhea was observed around 30 days after administration of sorafenib; we determined that it was an adverse event to sorafenib and suspended sorafenib for 21 days for recovery of diarrhea, and thus, resumed sorafenib 400 mg/day from day 99. Diarrhea did not recur thereafter, and we continued sorafenib therapy.

**Fig. 6:**
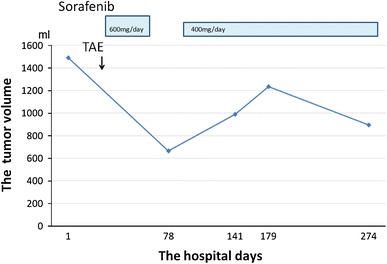
Changes in the size of hemangioma were determined by volumetry. The tumor volume was 1,492 mL before treatment. Sorafenib (600 mg/day) reduced the tumor volume to 665 mL on hospital day 78. While sorafenib therapy was suspended due to an adverse event, the tumor began to grow. After resuming sorafenib therapy at 400 mg/day, the tumor shrank again to a volume of 896 mL on hospital day 274

Inner tumor volume was measured by outline manual tracing with a three dimension workstation (manufactured by TeraRecon, Inc., Tokyo, Japan) axial image of 5 mm sliced helical abdominal CT during sorafenib treatment. Then it was converted to a three dimension volume rendering image. Furthermore, we determined the superficial measure of the hepatic hemangioma at each slice configured threshold for visualized target region. Tumor volume was autometrically calculated by multiplying these surface areas by product depth slice. Manual tracing and configuring threshold were executed by a single radiation technologist. At that time, the circumstances of image, threshold, and so on were secured under certain conditions. We presented changes of tumor size measured in this method (Fig. 6). It was 1492 mL at baseline; decreased to 665 mL at day 78 after administrating sorafenib 600 mg/day. It increased due to the interruption of treatment by the adverse reaction to sorafenib, though it decreased again to 896 mL at day 274 after resumption of sorafenib 400 mg/day.

During treatment, we measured concentration of peripheral VEGF; 23 pg/mL at diagnosis, 77 pg/mL at day 64, 60 pg/mL at day 78 and 59 pg/mL at day 232, all of these results were within normal range. It seems lack of utility for diagnosis and evaluation of treatment efficacy.

The shrinkage of the hepatic tumor, although possibly induced by the effect of TAE, is considered to be primarily due to the effect of treatment with sorafenib, because (1) the blood supply to the inside of the tumor was not obviously reduced on CT scans obtained after TAE; (2) other tumors in the right hepatic lobe, where the effect of TAE was considered unlikely to reach also shrank; and (3) the tumor, which was growing during suspension of sorafenib therapy, shrank again after resuming the treatment.

## Discussion

The incidence of cavernous hepatic hemangiomas is estimated to be 1–2 % [1, 2], and hemangiomas are incidentally discovered on abdominal ultrasound, CT, or MRI studies in many cases. Many hepatic hemangiomas grow slowly and remain asymptomatic in most cases.

Hepatic hemangiomas, if associated with symptoms, should be subject to intervention. A lesion of more than 4 cm in diameter is called a giant hepatic hemangioma, which causes abdominal symptoms, such as pain, nausea, anorexia, and a feeling of abdominal distention [3–5]. A hepatic hemangioma should also be treated if the patient concurrently develops, albeit in rare cases, hemorrhage, abscess, heart failure, jaundice, or Kasabach–Merritt syndrome, or if it suddenly ruptures or carries a high risk of rupture [6].

In this case, it was indicated for treatment based on increasing tumor size, cause of abdomen enlarged feeling and risk of rupture because tumor existed on surface of liver with subcapsular ecchymoma.

There are many reports that surgical resection is effective for the treatment of hepatic hemangiomas [7–11]. As a method of hepatic resection, segmental resection is usually selected if a hemangioma is localized in a single segment, and enucleation is selected if it is localized in the center of the liver [17–19].

In our case, since the tumor was large, involved both lobes of the liver, and the liver tissue remaining after resection would be too small to function, we did not select surgical resection.

There have also been reports on treatments other than surgical resection for hepatic hemangiomas, including radiation [20], ligation of hepatic artery [21], TAE [22, 23], and liver transplantation [24]; however, the effects of these treatments are limited, and the frequency of procedural complications is high [10].

Recently, a case of hepatic hemangioma shrunk by bevacizumab has been reported [14]. Bevacizumab is an anti-VEGF monoclonal antibody and is reported to have antitumor activity against HCC, ovarian cancer, renal cell cancer, pancreatic cancer, and soft tissue sarcoma when it is administered alone or in combination with chemotherapy [25].

Although the pathogenesis of cavernous hemangioma has not been elucidated, there is a theory that abnormal angiogenesis induced by increases in angiogenic factors, such as VEGF and matrix metalloproteinases (MMPs), and down-regulation of anti-angiogenic factors leads to the development of hemangiomas [12, 13]. Zhang et al. [26] discovered that the expression of VEGF-A, a member of the VEGF family, is increased in cavernous hepatic hemangiomas, as compared with hepatic sinusoidal epithelial cells, and reported that the increased expression of VEGF-A leads to an increased angiogenic activity in hemangiomas.

These findings suggest that bevacizumab exerts an antitumor effect on hemangiomas through its inhibition of VEGF [14], and that VEGF inhibitors may be effective for the treatment of hemangiomas.

Sorafenib is a multi-kinase inhibitor targeting Raf kinases in the downstream of the epidermal growth factor receptor (EGFR), VEGF receptors 1, 2, and 3, platelet-derived growth factor receptor-*β*, etc. [27]. Sorafenib has been reported to have antitumor effect on various solid tumors [28–30] and has also been demonstrated to be effective against HCC [31–33]. The drug is now widely used in the field of clinical oncology.

In addition, there are some reports on the use of sorafenib for the treatment of soft tissue sarcoma [34], and reports on sorafenib administered to patients with vascular tumors, including angiosarcoma, epithelioid hemangioendothelioma, and hemangiopericytoma/solitary fibrous tumor, are especially noteworthy [35].

These findings suggested that sorafenib might be effective for the treatment of hepatic hemangiomas, and we therefore decided to administer sorafenib to our patient.

As we described in his clinical course, the hemangioma shrank after administration of sorafenib, which was demonstrated by quantitative volumetric assessment on CT scans. On physical examination, a reduction in the size of the enlarged liver was noted, and the patient himself noticed alleviation of the symptoms.

By what mechanism did sorafenib exert a tumor-shrinking effect on the hemangioma? Since neither tumor biopsy specimens nor tumor resection specimens were obtained, we did not perform immunohistological evaluation of VEGF. Although we measured serum VEGF concentration before treatment, the baseline VEGF concentration was not high. Accordingly, it is not clear whether tumor shrinking was contributed to the VEGF inhibiting effect of sorafenib.

In summary, we have reported the first case of a man with a giant hepatic hemangioma in which sorafenib was effective, and suggested that sorafenib is a promising treatment option for such patients.
